# An update on the pathological classification of breast cancer

**DOI:** 10.1111/his.14786

**Published:** 2022-12-08

**Authors:** Emad A Rakha, Gary M Tse, Cecily M Quinn

**Affiliations:** ^1^ Translational Medical Sciences Unit, School of Medicine University of Nottingham Nottingham UK; ^2^ Department of Cellular Pathology Nottingham University Hospitals NHS Trust, Nottingham City Hospital Nottingham Nottingham UK; ^3^ Department of Anatomical and Cellular Pathology Prince of Wales Hospital, The Chinese University of Hong Kong Ngan Shing Street Shatin NT Hong Kong SAR; ^4^ Department of Histopathology St. Vincent's University Hospital Dublin Ireland

**Keywords:** breast cancer, classification, clinical, differentiation, grade, molecular, outcome, stage

## Abstract

Breast cancer (BC) is a heterogeneous disease, encompassing a diverse spectrum of tumours with varying morphological, biological, and clinical phenotypes. Although tumours may show phenotypic overlap, they often display different biological behaviour and response to therapy. Advances in high‐throughput molecular techniques and bioinformatics have contributed to improved understanding of BC biology and refinement of molecular taxonomy with the identification of specific molecular subclasses. Although the traditional pathological morphological classification of BC is of paramount importance and provides diagnostic and prognostic information, current interest focusses on the use of a single gene and multigene assays to stratify BC into distinct groups to guide decisions on systemic therapy. This review considers approaches to the classification of BC, including their limitations, and with particular emphasis on the fundamental role of morphology in establishing an accurate diagnosis of primary invasive carcinoma of breast origin. This forms the basis for further morphological characterization and for all other approaches to BC classification that are used to provide prognostic and therapeutic predictive information.

## Graphical abstract

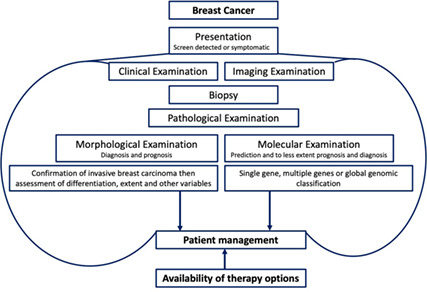

## Background

Breast cancer (BC) comprises a heterogeneous group of tumours that displays marked variation in clinical presentation, morphology, molecular features, biological behaviour, and response to therapy. Despite major advances in our understanding and management of BC, BC remains a major public health problem and continues to pose significant challenges worldwide.

The diagnosis of BC dates back 3500 years,^1^ when it was classified according to visible signs and symptoms of the disease. The realization, in the mid‐18th century, that cancer is a local disease that progresses in stages rather than a *de novo* systemic disease led to proposals of early surgical removal of a breast tumour before it had spread to the axillary lymph nodes. Mastectomy continued to be the mainstay of treatment of BC until the second half of the 20^th^ century. Until then, the pathological examination of excised breast tissue was primarily an exercise in confirming the diagnosis of BC without pathological prognostic stratification of the disease.^2^, ^3^

The 1960s heralded the discovery of the biological significance of hormone receptor status in BC leading to the approval of the anti‐oestrogen drug Tamoxifen. The 1980s witnessed the introduction of population based mammographic screening.^4^ The refined criteria for histological assessment of tumour type^5^, ^6^ and grade^7^, ^8^ and their prognostic significance, in addition to the prognostic importance of other pathological variables,^9^, ^10^ were also published. In the 1990s, taxanes and capecitabine, important chemotherapeutic drugs, were approved for the adjuvant management of BC. During that decade, sentinel lymph node biopsy was introduced as an alternative to full axillary lymph node clearance for the staging of BC, and the specific inherited mutations in the tumour suppressor genes, *BRCA*1 and *BRCA*2,^1^ were identified. Towards the end of 1990s, the first targeted anti‐HER2 drug, trastuzumab (Herceptin) was approved for the management of metastatic BC. These changes were accompanied by a significant improvement in the pathological diagnostic and prognostic classification of BC, which included detailed histomorphological assessment in addition to evaluation of the hormone receptor and human epidermal growth factor receptor 2 (HER2).

In the early 2000s, the concept of the molecular classification of BC was introduced with the recognition of intrinsic molecular subtypes and the development of multigene signatures, representing a significant advance in our understanding of BC. Despite the importance of the well‐established morphological prognostic variables in BC, this focus has dominated BC research in the last two decades, facilitated by the development of high‐throughput molecular techniques, such as microarrays and next‐generation sequencing, and is rapidly expanding in response to the increasing availability of targeted therapy and the move towards precision and personalized medicine.

These important and continuously evolving advances in our understanding of the biology and clinical management of BC, together with improved BC detection and an appreciation of its significant heterogeneity, have emphasized the importance of a patient‐focussed pathological classification with biological and clinical relevance (Table 1). This includes complex and expanding systems of classification that are based on the diagnosis, prognostic evaluation, and predictive stratification in addition to the stratification of BC into other clinically relevant groups to assist disease monitoring, genetic counselling, and risk factor assessment.

Following initial diagnosis and confirmation of a primary breast tumour, further histological classification is typically based on the type and degree of differentiation (tumour type and histological grade), by examination of haematoxylin and eosin (H&E)‐stained slides, taking account the gross findings and supported by special stains, immunohistochemistry (IHC), and other molecular assays, as required. The assessment of prognostic markers and other parameters such as tumour stage, lymphovascular invasion (LVI), margin status, and the identification of coexisting and precursor lesions, guide further management^11^ and complement the diagnostic classification of BC. For instance, the demonstration of LVI or lymph node metastasis confirm the invasive nature of the tumour in addition to being of prognostic value. Similarly, documentation of the molecular features of BC, which are primarily used for predictive purposes, may also have diagnostic and prognostic value. In this review we address the pathological classification systems in BC with emphasis on morphological and molecular classification. Clinical and epidemiological classification are beyond the scope of this review.

**Table 1:** Main classification systems of breast cancer

Classifier	Variables
Presentation	• Detection (Screen‐detected versus symptomatic). • Stage (Early stage, locally advanced, or metastatic). • *Signs and symptoms (inflammatory BC, lump size, consistency, shape, and fixation, skin and nipple changes, axilla, laterality, and focality) • Menopausal status (premenopausal versus postmenopausal). • Others: gender, age, ethnic origin, family history.
Imaging	Mass shape, margin, depth, and site, breast composition, calcification, axillary findings, laterality
Pathological	
*Morphological classification* (mainly diagnostic and prognostic)	
Tumour differentiation	
Tumour type:	Several tumour types (currently at least 18 tumour types) are described and some types include multiple variants^20^ based on the combination of cytological, architecture features, and secretory activity and stromal features
Tumour grade	Three grades^19^ based on the degree of differentiation and similarity to TDLUs
Disease extent	
Tumour stage	Invasive tumour size, infiltration of other tissues, lymph node status, and assessment of lesions at distant sites
Other factors	
	Lymphovascular invasion (present or absent), presence and extent of the *in situ* lesions (DCIS), stromal features such as TILs, Paget's disease, focality, bilaterality, and excision status
*Molecular classification* (mainly predictive but can provide diagnostic and prognostic value)	
Single gene classifier	Oestrogen receptor and HER2 are the most important classifiers to guide treatment decision with the addition of PDL1 Other markers include progesterone receptor (PR), KI67 as prognostic markers Familial predisposition genes such as BRCA1, BRCA2, and PALB2.
Multiple gene classifier	Multigene prognostic signatures are composed of multiple genes assessed together to assess risk in certain BC groups mainly the luminal class.
Global gene expression and genomic classification	• Intrinsic molecular subtypes including luminal, basal and HER2 enriched. • Mutation signatures • Integrated class classification based on a combination of transcriptomic and genomic (e.g. gene copy number) classification
Therapy classification	• Systemic therapy naïve versus treated patients. • Neoadjuvant therapy treated versus adjuvant treated patients. • Type of therapy (hormone, cytotoxic, targeted, or immunotherapy). • Line of therapy (first‐line therapy versus second‐ or third‐line)

### Morphological classification of BC

Over the last few decades, the classification of BC has moved from a simple pathological diagnosis, based on confirmation of cancer and a description of type such as “adenocarcinoma of the breast” or “scirrhous breast carcinoma”, to comprehensive synoptic reports that now include more than 20 core items in addition to a series of noncore items. Several national organizations, including the United Kingdom Royal College of Pathologists (RCPath) and the College of American Pathologists (CAP), have published BC datasets and guideline recommendations to help pathologists involved in reporting BC to improve concordance and so to enhance patient care.^12^, ^13^, ^14^ In further recognition of the importance of pathology reports in providing the fundamental information required for the management of BC patients, the International Collaboration on Cancer Reporting (ICCR) was founded by major pathology organizations from around the world to produce internationally standardized and evidence‐based datasets to improve cancer patient outcomes worldwide and to advance international benchmarking in cancer management (http://www.iccr‐cancer.org/).

#### Pathological staging classification

BC stage is the most important prognostic variable. Early‐stage BC has 10‐year survival rates of over 90%. In contrast, metastatic BC, which accounts for approximately 6–7% of *de novo* presentations and develops in ~30% of women with early‐stage BC at diagnosis, is associated with 5‐year relative survival rates of ~25%, and a median overall survival (OS) of ~2 years.^15^ This difference in the outcome is observed regardless of the histological type, grade, or molecular features of the disease. Differences in outcome, to a lesser degree, are also observed with local versus regional disease extent. Despite the importance of the clinical and radiological staging of BC, pathological staging remains the gold standard and provides detailed staging information including confirmation of primary tumour size, infiltration of local structures, the presence of lymph node metastases, and estimation of nodal disease burden.

Several staging classifications have been published, acknowledging the importance and the clinical relevance of BC disease extent. The Tumour Node Metastasis (TNM) system, currently the most widely used staging system, classifies extent according to the primary tumour (T) size, nodal (N) involvement, and metastasis (M) based on clinical and pathological evaluations. In addition, the TNM recognizes some specific clinical presentations including inflammatory carcinoma (T4d) and skin ulceration (T4b), as these have distinct BC behavioural patterns, and the presence and pattern of local spread including chest wall infiltration (T4a) and the presence of ipsilateral macroscopic satellite nodules (T4b). The TNM system was developed and is maintained by the Union for International Cancer Control (UICC) and is also used by the American Joint Committee on Cancer (AJCC).^16^ This staging classification system is updated on a regular basis and is used by pathologists worldwide.

The latest 8^th^ edition of the AJCC Staging Manual, in addition to retaining traditional anatomic staging, recognized the importance of the biological and molecular variables. It introduced a prognostic staging system, which incorporates tumour grade, hormone receptors (oestrogen receptor [ER], and progesterone receptor [PR]) and oncogene status (HER2), further modified to include multigene panel results in a subset of patients to amend the anatomical stage.^16^ This new approach, aimed to better reflect BC prognosis, will result in the restaging of some patients, but has limited applications in some scenarios such as triple‐negative and advanced‐stage BC.^17^ In many parts of the world, biological markers and multigene panels are not routinely available, limiting the worldwide applicability of such a staging system. Many countries, including the UK, continue to rely on the anatomical staging system.

The Nottingham Prognostic Index (NPI^18^), the first BC prognostic staging system to be developed, based on the lymph node stage (1–3), histological grade (1–3),^19^ and the primary tumour size, is still used in many centres and provides one of the most cost‐efficient and easy‐to‐use prognostic tools in BC. BC staging is primarily used to risk stratify patients for consideration for therapy, rather than to determine the specific type of therapy. It is also important to note that therapy may modify the behaviour of tumours and the original estimated risk may change with treatment. Therefore, the risk classification of BC includes two predicted estimates: the original therapy‐naïve risk and the posttreatment risk.

### Classification based on tumour differentiation

Despite the clinical importance of BC staging, the histomorphological classification plays the key role in BC diagnosis and provides the basis for all other classification systems. This classification system relies heavily on the performance and expertise of pathologists, with limited input from molecular tests. Daily challenges include the distinction between *in situ* and invasive disease, tumour typing and grading, and the distinction of primary breast cancers from their mimics. No staging or molecular classification system is of value without the histological confirmation of BC and the accuracy of diagnosis.

The histological diagnosis of BC is based on the evaluation of certain features, individually and in combination, including the cytological and architectural features of the proliferating cells, tumour‐associated stroma, demonstration of the presence or absence of myoepithelial cells at the epithelial stroma interface, using H&E‐stained slides, supported by the use of IHC and other molecular assays in certain situations. The confirmation of invasive BC of primary breast origin is followed by the assessment of tumour differentiation. Differentiation in BC can be measured morphologically using tumour histological grade and type. Histological grade, which measures the similarity of a tumour to the normal breast terminal duct lobular units (TDLUs),^20^ reflects the degree of differentiation, whereas tumour type reflects the type of differentiation.^19^, ^21^ BCs are graded using the Nottingham grading system, which involves the semiquantitative evaluation of three important biology‐dependent morphological features: (i) degree of tubule or gland formation, (ii) nuclear pleomorphism, and (iii) mitotic count.^22^ Although the Nottingham tumour grade is prognostically relevant in all BC histological subtypes, some BCs have a certain histological grade determined by their type, e.g. tubular, invasive cribriform carcinoma, low‐grade adenosquamous carcinoma, and fibromatosis like metaplastic carcinoma are grade 1 by definition, whereas IBC with medullary features and basal‐like BC are grade 3 tumours. Most BCs, including invasive ductal carcinoma – no special type (IBC‐NST), lobular, mucinous, and metaplastic carcinoma show a spectrum of histological grade while maintaining specific tumour type characteristics.

Tumour type is determined according to specific tumour characteristics, including cytological features, tumour growth pattern and architecture, secretory activity, and stromal features. Identification of tumour type is important for diagnosis and confirmation of primary breast origin. Although some tumour types are associated with distinct clinical behavioural patterns, the individual tumour type is of variable prognostic and predictive value. This is mainly due to the presence of several histological types of BC and the existence of several variants of the more common types, e.g. IBC‐NST, lobular and metaplastic carcinomas. IBC‐NST accounts for most primary BCs (60–75%), while some special type tumours comprise <2% of all BCs. Despite this, the prognostic significance of tumour type can be improved if the tumour types are grouped into prognostic groups (Table 2), combined with the tumour grade,^5^, ^22^, ^23^, ^24^ and ideally with receptor status and tumour size. HER2‐positive and triple‐negative tumours are likely to have a poorer prognosis compared to ER‐positive tumours of the same type and stage. Very small tumours (<5 mm) typically have a very good prognosis, regardless of the tumour type.^25^ Some tumour types such as lobular and metaplastic carcinomas are likely to show less response to chemotherapy compared with IBC‐NST.

**Table 2:** Prognostic tumour type groups

Prognostic groups	Types
Very indolent (excellent prognosis similar to locally infiltrative lesions with limited metastatic potential)	Pure low‐grade adenosquamous carcinoma, pure fibromatosis like metaplastic carcinoma, pure low‐grade mucoepidermoid, adenoid cystic and secretory carcinomas^81^, * Other special type tumours which include encapsulated and solid papillary carcinomas that lack myoepithelial cells but staged as *in situ* disease (pTis), and other lesions such as atypical adenomyoepithelioma and malignant adenomyoepithelioma *in situ*.^82^
Excellent prognosis group (low metastatic potential. Mainly lymph node metastasis)	Pure tubular and invasive cribriform carcinoma of limited size (<3 cm)*
Good prognosis group	Grade 1 invasive lobular, mucinous, invasive papillary and IBC‐NST, and tubulolobular carcinoma.
Moderate prognosis group	Grade 2 IBC‐NST, and invasive lobular carcinoma classical type.
Poor prognosis group	High grade IBC‐NST, solid and other high‐grade invasive lobular carcinoma, high‐grade matrix producing and squamous cell metaplastic carcinomas.
Very poor prognosis group	High grade spindle cell metaplastic carcinoma, small cell carcinoma and high‐grade triple‐negative IBC‐NST of large size.

* These tumours should be small (<3 cm). During tumourigenesis, the cancer cells undergo replication and mutation, thereby increasing the tumour size is often associated with increasing invasiveness of the tumour.^83^ Larger size tumours are likely to have different tumour components and the behaviour is likely to relate to the other (more aggressive) carcinoma component. The basaloid and solid variant of adenoid cystic carcinoma is more aggressive. Also, some secretory carcinomas in older patients may behave less indolently.

Tumour typing is a dynamic process and new entities are described and old entities are renamed or combined with other tumour types. Some pathologists consider tumours with certain features as new or distinct entities (‘splitting’), while others will link the features to a more common and well‐established tumour type and consider these tumours as variants of the main tumour type (‘lumping’). The recognition of new entities usually starts with the publication of case reports and/or case series reporting on the histological features of certain breast lesions and the behaviour of such lesions. In contrast, the recognition that a rare tumour type overlaps histologically with another more common tumour type and the lack of distinct clinical value in specific recognition may result in grouping these tumours together, best exemplified by the recent inclusion of medullary carcinoma into the IBC‐NST category. Similarly, basal‐like carcinoma was proposed as a special tumour type following the description of the basal‐like/triple‐negative molecular subtype. It was subsequently shown that these tumours showed morphological overlap with other high‐grade IBC‐NST tumours and that some basal‐like tumours defined using molecular assays did not display the same histological features.

The World Health Organization (WHO) series on the Classification of Tumours (also known as the WHO Blue Books) is regarded as the gold standard for the diagnosis of tumours and provide indispensable international standards for classification of breast tumours worldwide.^20^ The 5^th^ edition (2019) WHO Classification of Breast Tumours continues to recognize several special types of BC, which together account for up to 25% of all invasive BCs.^20^ Knowledge of these special types helps pathologists to recognize that a tumour is of primary breast origin and may provide clinically relevant information. For example, a diagnosis of invasive lobular carcinoma (ILC) on core needle biopsy (CNB) usually leads to further preoperative imaging due to the increased incidence of multifocality and bilaterality. Invasive lobular carcinoma is also less likely to respond to chemotherapy, which is important in patient selection for neoadjuvant chemotherapy. Metaplastic carcinoma is generally associated with a poor prognosis and a limited response to neoadjuvant chemotherapy.

**Figure 1:**
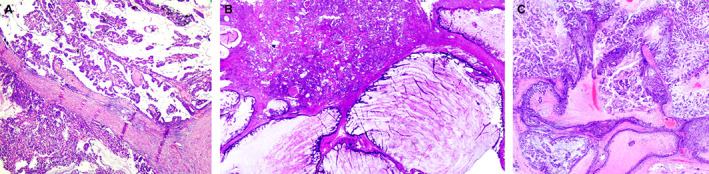
A case of mucinous cystadenocarcinoma featuring complex papillary growth pattern (**A**) and prominent cystic spaces (**B**) with mucinous differentiation (**C**) mimicking mucinous cystadenocarcinoma of the ovary and some other organs.

**Figure 2:**
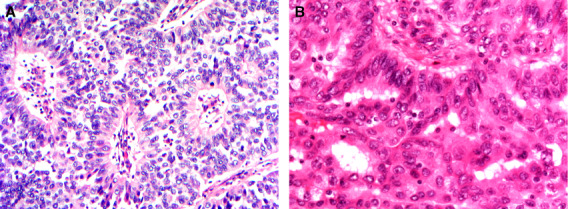
A case of tall cell carcinoma with reversed polarity featuring nuclei placed away from the basal border (**A**), nuclear groove and nuclear overlapping (**B**) similar to the tall cell variant of papillary thyroid carcinoma.

The 5^th^ edition WHO working group^20^ introduced some changes concerning tumour typing, reflecting not only improved understanding of tumour biology but also challenges in achieving diagnostic concordance. Some rare tumours, e.g. tall cell carcinoma of breast with reversed polarity and mucinous cystadenocarcinoma are now recognized as special type BCs (Figures 1 and 2). Other rare tumour types including salivary gland‐like tumours, apocrine carcinoma, and invasive papillary carcinoma continue to be recognized as special types with distinct molecular and clinical features. Knowledge of these entities will avoid misclassification as metastatic tumour and provide information on likely biological behaviour. However, other tumour types, considered to represent end of differentiation of IBC‐NST, have been reassigned to the IBC‐NST category with a designation of special morphological pattern including medullary/medullary‐like carcinomas, glycogen‐rich, lipid‐rich, sebaceous, and oncocytic carcinomas. In addition to morphological overlap with IBC‐NST, the prognosis of these tumours does not differ from grade matched classical IBC‐NST.

A particular challenge encountered by pathologists in breast tumour typing is the recognition of tumours that are associated with biological behaviour like that observed in *in situ* breast carcinoma. The distinction between *in situ* and invasive carcinoma may be blurred and lacks a strong evidence base. The approach to categorisation varies depending on the specific tumour, resulting in a lack of diagnostic concordance among pathologists. Examples include solid and encapsulated papillary carcinomas that lack peripheral myoepithelial cells. These tumours are currently designated as *in situ* tumours, although they are likely to represent indolent invasive tumours with low metastatic potential.^26^, ^27^ In contrast, pure low‐grade adenosquamous carcinoma and pure low‐grade mucoepidermoid carcinoma of the breast do not demonstrate metastatic potential, but are managed as invasive tumours. Although low‐grade adenosquamous carcinoma and syringomatous tumour of the nipple share histological and molecular features, the first is designated as carcinoma, while the latter is considered benign. Neuroendocrine (NE) tumours of the breast have long been a source of confusion regarding cell of origin, terminology, diagnostic criteria, and the lack of distinction between invasive and *in situ* lesions with consequent management implications. Breast NE neoplasms (NENs) are currently described either as NE tumours or NE carcinoma, although all represent carcinomas.^20^ Further refinement of the classification of these breast tumours is needed to improve their diagnostic reproducibility and consolidate the clinical significance of each diagnosis.^28^

ILC is often considered to be a single tumour type characterized by loss of function of the cell adhesion protein, E‐cadherin, with subsequent cell dyscohesion but comprises a spectrum of tumours with different histological features and clinical behaviour. The classical variant is the most common subtype, typically Nottingham grade 2, and shows a distinct clinical behaviour.^29^ Most of the data on the clinical behaviour of ILC are derived from this variant, with less available information on the clinical behaviour of other variants. The solid ILC variant is characterized by a solid growth pattern, shows high mitotic activity, and may be associated with aggressive clinical behaviour. The pleomorphic ILC variant is characterized by high‐grade cytological features and a poor prognosis, whereas the alveolar and tubulo‐lobular variants are characterized by a good prognosis.^30^ Metaplastic breast carcinoma (MBC) comprises a heterogeneous group of tumours with a range of histological features and clinical behaviour, but is also sometimes considered by pathologists and clinicians to represent a single tumour type. MBC subtypes reflect variable differentiation pathways that are distinct from the adenocarcinoma differentiation pathways. Two main differentiation pathways are recognized in MBC: squamous and mesenchymal, the latter including spindle cell and matrix producing differentiation. MBC includes indolent low‐grade tumours such as fibromatosis‐like spindle cell MBC and low‐grade adenosquamous carcinoma and the aggressive high‐grade spindle cell MBC and high‐grade adenosquamous carcinoma.^31^, ^32^ Intermediate nuclear grade (grade 2) spindle cell metaplastic carcinoma can rarely be encountered, and these tumours have intermediate risk between the fibromatosis like and the high‐grade spindle cell MBC. In practice, we observe two main types of MBC: (1) tumours with metaplastic and adenocarcinomatous components with morphological and/or biomarker overlap, considered as MBC regardless of the percentage of the tumour occupied by metaplastic elements. In these tumours, the adenocarcinomatous component are typically high grade and shows a triple‐negative phenotype and the transition between the two components is gradual, and (2) tumours with a distinct metaplastic component (e.g. spindle cell, matrix producing or squamous) that may coexist with IBC‐NST or another special type component. The second component (IBC‐NST or special type) in these tumours may show receptor positivity and there is a clear distinction between such a component and the metaplastic tumour component. Although not specified in the WHO book, these tumours can be regarded as pure MBC if the metaplastic component exceeds 90%, and as mixed metaplastic and NST tumours if the metaplastic component accounts for >10% and < 90% of the tumour. In general, the presence of a high‐grade metaplastic element is associated with aggressive clinical behaviour and is likely to drive the behaviour of the mixed tumours regardless of percentage. Therefore, it is advised that the presence of high‐grade metaplastic component in mixed tumours to be stated even if it is a minor component.

Conventional IBC‐NST carcinoma occasionally contains minor components of other special type BCs.^33^ When the special type component forms a recognizable proportion of the tumour (10–90%), the term mixed carcinoma is used.^20^ The concordance of classifying mixed BC in clinical practice is low,^34^ which may reflect the difficulty in distinguishing the special type from the nonspecial component.

## Molecular classification

There are several lines of evidence to suggest that the clinical and morphological parameters currently available are insufficient to fully reflect the biological heterogeneity of BC, and that tumours with similar morphology and stage vary in clinical behaviour and response to therapy. Moreover, the widespread use of mammographic screening, improved understanding of the nature and biology of BC, and the increasing array of systemic therapy options (hormone, chemotherapy, anti‐HER2, and other targeted therapy and immunotherapy)^35^ further emphasizes the need to utilize molecular prognostic and predictive markers. These are primary tumour molecular characteristics that can be used to determine tumour behaviour (prognostic) and response to specific therapy (predictive).^36^ Molecular classifiers include genes and their products (RNA and protein) that can be assessed individually or in consort (e.g. molecular profiling).

### Single gene classifiers

Of the individual molecular markers, ER and HER2 have proven of predictive and prognostic value. ER and HER2 status, which are an essential part of the diagnostic workup of all BC patients, are determined using standardized techniques according to well‐defined published guidelines.^12^, ^37^, ^38^, ^39^ Clinically, all invasive BCs are grouped into following biomarker‐defined subtypes/groups for treatment purposes: (1) ER‐positive, HER2‐negative, (2) ER‐positive, HER2‐positive, (3) ER‐negative, HER2‐positive, and (4) ER‐negative, HER2‐negative cancers.^20^ It is currently recognized that the main consideration for adjuvant treatment of BC is potential tumour endocrine responsiveness. Adjuvant hormone therapy accounts for almost two‐thirds of the overall benefit of adjuvant therapy in patients with ER‐positive BC. Tumour ER expression predicts response to hormone therapy in 30–60%, while ER‐negativity, which accounts for 20–30% of breast cancer, identifies a population of patients who will not benefit from endocrine therapy.^40^, ^41^, ^42^, ^43^ Approximately 30% of ER‐positive tumours are PR‐negative and ER+/PR− tumours are generally less responsive than ER+/PR+ tumours.^44^, ^45^, ^46^ Lack of PR expression in ER‐positive tumours may be a surrogate marker of aberrant growth factor signalling that could contribute to tamoxifen resistance. Multiple studies have provided evidence for the prognostic and predictive importance of PR in BC.^46^, ^47^, ^48^, ^49^ TheER−/PR+ phenotype is rare but exists and is not purely a staining artefact (ER false‐negative or PR false‐positive on CNB). Earlier studies have reported 10% or more of BC show ER−/PR+^50^; however, recent data indicate that this phenotype comprises ~1−2%.^51^ It is our experience that few cases (<1%) remain as biologically relevant ER−/PR+ phenotype (convincingly PR‐positive (moderate to strong nuclear staining in >10% of the tumour cells) and ER‐negative (<1%) when the staining is repeated on CNB or the excision specimens. It is our opinion that weak PR staining in 1–10% of the tumour cells is unlikely to have clinical or biological significance on ER− BC in terms of response to therapy, or clinical behaviour. The outcome of ER−/PR+ BC is not clear, but it is likely worse than ER+/PR+ tumours.^52^, ^53^

Although for management purpose, a cutoff of 1% is used to define ER positivity and define eligibility for endocrine therapy, the level of ER expression in BC is variable (the intensity of expression varies from weak to strong and the frequency of positive cell varies from 1% to 100%). This has prognostic significance in terms of better outcome and response to endocrine therapy in BC, showing strong diffuse nuclear expression. To categorize patients into prognostic groups, multiple scoring systems are developed including the Quick score, Allred Score, and H. score, which consider a combination of intensity of staining and the percentage of positive cells to produce a score that can be categorized into subgroups of prognostic significance.^54^

Use of anti‐HER2 therapy is based on HER2 status, determined using IHC and/or *in situ* hybridisation (ISH) studies, combined with risk stratification.^55^ Amplification of *HER2* gene occurs in 12–20% of BCs and more than half (~55%) of these tumours are ER‐negative.^56^, ^57^ Numerous studies have shown that *HER2* gene amplification/protein overexpression is a predictor of poor prognosis and response to certain types of chemotherapy.^58^, ^59^, ^60^ ER and HER2 are assessed in daily practice to provide information on response to endocrine therapy and anti‐HER2 targeted therapy, respectively. However, expression of these biomarkers overlaps and their prognostic and predictive value can be improved by using them in combination^61^ and also in combination with PR status and the proliferation marker KI67.^62^ Most IHC studies have used a combination of ER, PR, and HER2 with or without KI67 as IHC surrogates to define the molecular classes initially identified by gene expression profiling (intrinsic subtypes). ER positivity is a surrogate for luminal class, HER2 expression for HER2‐positive tumours, and the triple‐negative (ER‐, PR‐, HER2‐) phenotype is used to define the basal‐like molecular class.^63^ Some other genes, which are assessed individually in BC, such as PR and KI67,^64^ have been shown to be of specific clinical utility in classifying luminal tumours as luminal A or B.^65^

More recently, PDL1 is being used as a predictive marker for potential response to immunotherapy. Other genes that are used to classify BC include *BRCA*1, *BRCA*2, and *PIK3C*A, the latter is also used to guide systemic treatment. It is likely that additional genes will be used to guide therapy decision‐making in the future.

### Multigene classifiers

The introduction of the concept that BC can be classified using global gene expression profiling in 2000^66^ (molecular taxonomy) has revolutionized BC research. The total gene expression pattern of a given sample is known as a gene expression profile, often referred to as a ‘signature’ or ‘portrait’. Most tumours display expression signatures/profiles that are unique and related to specific biological features.^67^, ^68^ Although the molecular classification system provides prognostic value and possible predictive information and has contributed to our current understanding of BC molecular complexity, its application in the clinical setting and influence on BC therapeutic decision‐making remains less than was anticipated. The added value of these studies over the routinely assessed products of individual genes, ER, HER2, PR, and KI67, is currently limited.

ER‐positive luminal and HER2‐positive tumours had been characterized before the advent of gene expression molecular taxonomy. The basal‐like group attracted particular attention as a novel class characterized by triple‐negative phenotype, poor outcome, and the generally similar molecular profile of these tumours that clustered together at the molecular level. Subsequent studies have, however, demonstrated several molecular subclasses in this basal/triple‐negative BC group, including luminal androgen receptor type tumours.^69^, ^70^ However, the clinical and therapeutic relevance of these subclasses and the additional value of performing androgen receptor and basal marker expression is not yet clear. Similarly, patients with HER2‐positive BC determined using IHC and/or ISH studies are likely to be offered in anti‐HER2 therapy, regardless of molecular portrait, while it is not current practice to offer this treatment to patients with HER‐2‐positive BC based on molecular portrait alone.

Clinical relevance needs to be considered and factored into any emerging classification system to ensure that patients are treated appropriately. Moreover, it remains unknown how many molecular subclasses exist and, more important, how many can be reliably identified with currently available technology. The four main molecular classes frequently reported may represent an oversimplification of a novel molecular classification system that does not greatly advance our knowledge of the likely biological behaviour of BC. BC has also been classified using integrated analysis of gene copy number (DNA) and gene expression using gene transcripts (RNA).^71^ Classification based on the expression of several genes using IHC and tissue microarrays may also help to identify key molecular classes.^72^

### Multigene signatures

In addition to the molecular classes, a few prognostic multigene signatures have been identified based on the differential expression of a selected set of genes in a specific subgroup of tumours. Multigene signatures include “prognostic gene signatures” that can predict outcome. Other gene signatures have been developed based on prediction of response to specific therapy and are used as predictive signatures.^73^, ^74^ A common character shared by all these signatures is the use of combinations of genes, rather than using single genes, to predict a certain outcome that appears to reflect the overall genetic derangements underlying the complex tumour biology. To date, these signatures have not replaced the currently used prognostic and predictive factors in the management of BC, but provide useful complementary information to traditional clinicopathological parameters in the clinically intermediate risk group of patients, in particular those with ER‐positive, HER2‐negative early‐stage BC. The prognostic value of these tests in ER‐negative and HER2‐positive BC remains limited.

### Classification based on mutational signatures

The BC genome is a record of the mutagenic activity that has occurred throughout the development of a tumour. The clinical significance of BC mutations includes not only driver gene mutations but also passenger mutational signatures, gene rearrangement, the imprints of DNA damage, and DNA repair processes.^75^, ^76^ The existence of mutational signatures in BC was first described utilizing more than 183 thousand substitutions in 21 whole BC genomes.^77^ This was followed by a large study exploring 560 BCs that identified a total of 12 substitution signatures from over 3,479 thousand mutations.^78^ Some of these signatures are variously associated with age at diagnosis, *BRCA*1/*BRCA*2 deficiency, the activity of the APOBEC cytidine deaminases, or with mismatch repair deficiency. Importantly, these mutational signatures do not appear to demonstrate specificity to BC subtype, whether classified by ER status or intrinsic molecular subtype. The mutational signatures not only include base substitutions, small insertions, and deletions (indels), but they have also extended to structural variation (genome rearrangement signatures).^78^ Rearrangements were classified into clustered (at specific loci reporting driver amplicons or simply at sites of chromothripsis^79^) or dispersed (equivalent number of rearrangements that are widely distributed throughout the genome), then divided according to rearrangement class (tandem duplication, deletion, inversion, or translocation) and size.^78^ Following this classification system six rearrangement signatures and seven major subgroups that exhibited distinct associations with other genomic, histological, gene expression, and clinical features were described.^76^

BC can also be classified based on the genetics of familial predisposition including the key predisposition genes, *BRCA*1, *BRCA*2, and *PALB*2, in addition to other genes based on penetrance and frequency in the population.^20^ There is increasing interest in classifying BC using multigene BC susceptibility and polygenic risk scores. Patients with germline and/or somatic mutations in *BRCA*1 and/or *BRCA*2, show sensitivity to poly (ADP‐ribose) polymerase (PARP) inhibitors.^80^

## Conclusion

BC is a heterogeneous disease that can be classified using several classification systems. These include clinical, imaging, and pathological morphological and molecular classifications, each with further subclassification systems. Knowledge of systems is likely to increase concordance of BC diagnosis and standardization of BC management. Although the clinical and molecular classification systems are important for determination of prognosis and prediction of response to therapy decisions, it is the pathological morphological diagnosis that is the foundation of other classification systems, used to confirm the diagnosis of malignancy, characterise as an invasive tumour of breast origin, and provide information on tumour type, grade, and other key prognostic variables. There is an increasing focus on the use of single genes, multiple genes, and global gene expression BC classifiers, which provide varying degrees of predictive information and act as companion diagnostics in the BC management workup. Applications of next‐generation sequencing to BC research are expanding and may change the way we understand and treat BC in the future. Despite the enormous amount of work that has been carried out to develop and refine BC molecular prognostic and predictive assays, this is still evolving. With the increasing use of more sophisticated molecular techniques, large amounts of data will continue to emerge, which could potentially lead to identification of novel therapeutic targets and allow more precise classification systems that can more accurately predict patient outcome and response to therapy.

## Acknowledegement

N/A

## Author Contributions

Emad Rakha, drafted the article, reviewed, and approved the final version. Cecily Quin reviewed and amended the article and approved the final version. Gary Tse reviewed and approved the final version of the article.

## Ethical Approval and Consent to Participate

N/A

## Funding

N/A

## Conflict of Interest

The author's have no conflicts of interest to declare.

## Data Availability

Data sharing is not applicable to this article as no new data were created or analysed in this study.
